# Parental and physician’s point-of-view towards antibiotic prescriptions and discharge conversations in the pediatric emergency department

**DOI:** 10.1186/s12887-022-03173-w

**Published:** 2022-03-10

**Authors:** Daphne Peeters, Lars M. A. van Scheppingen, Gertjan J. A. Driessen, Lilly M. Verhagen

**Affiliations:** 1 Department of Pediatrics, Juliana Children’s Hospital, The Hague, The Netherlands; 2 Department of Pediatrics, Maastricht University Medical Center+ (MUMC+), Maastricht, the Netherlands; 3 Department of Pediatric Infectious Diseases and Immunology, Wilhelmina Children’s Hospital, University Medical Center Utrecht, Utrecht, The Netherlands; 4 Department of Pediatric Infectious Diseases and Immunology, Radboudumc, Nijmegen, The Netherlands

**Keywords:** Parental opinion, Children, Infections, Antibiotics, Emergency department

## Abstract

**Background:**

Within Europe, the Netherlands has one of the lowest antibiotic consumption rates. We aimed to gain insight into attitudes of Dutch physicians and parents towards information provided during discharge conversations in the emergency department (ED) and towards antibiotic use in children, in order to obtain information on the assumptions and beliefs that underlie a practice of low prescription rates.

**Methods:**

Discharge conversations of 70 children presenting with an infectious disease at the ED were observed. After 7–10 days, 55 parents were called for a semi-structured interview. In addition, 29 pediatricians and pediatric residents completed a questionnaire on their prescription behaviour.

**Results:**

Concerns about (recognizing) the severity of their child’s infection was parents’ main motivation to seek help. Both pediatricians and parents reported a general reluctance towards antibiotic use. While pediatricians took appropriateness based on indication and the risk of antimicrobial resistance development into account when considering antibiotic treatment, a thorough medical assessment was deemed more important for Dutch parents than any type of therapeutic treatment, including antibiotics. The topic most often discussed during the discharge conversations was safety netting instructions (in 86%), which were discussed more often during discharge conversations with parents of children that did not receive antibiotic treatment (91% versus 69%).

**Conclusion:**

Dutch pediatricians and parents are both reluctant to use antibiotics for uncomplicated infections in children, but for different reasons. The emphasis of discharge conversations was on safety netting instructions, which seems to be an alternative for (early) antibiotic use in our setting and may guide overuse prevention strategies in settings where antibiotic overuse is more common.

**Supplementary Information:**

The online version contains supplementary material available at 10.1186/s12887-022-03173-w.

## Background

Antibiotic prescription practices show large inter-country variation. The overall worldwide prescription rate is high with an increasing prevalence of antimicrobial resistance (AMR) as an unwanted outcome [1, 2]. One of the main patient groups who often receive antibiotic treatment are children with infectious diseases. Approximately 23–32% of children aged 0 to 17 years presenting at emergency care facilities receive antibiotic treatment. Most of these antibiotic prescriptions are for children with acute respiratory tract infections [3, 4]. Up to 32% of all children residing in the USA who received antibiotics after their visit to the emergency department (ED) from 2009 to 2014 had a diagnosis for which antibiotics are generally not indicated [4]. These numbers indicate that annually millions of children unnecessarily receive antibiotics when visiting the ED. However, in some countries, such as The Netherlands, antibiotic prescription rates are relatively low. In fact, existing literature shows that prescription rates in The Netherlands are the lowest of Europe and among the lowest in the world [2, 5].

An ED consultation generally contains multiple elements: discussing the child’s history and current complaints, a physical examination, diagnostic tests and a management plan. In case of a suspected infection, the pediatrician has to consider the potential indication for antibiotic treatment. This consideration is influenced by multiple variables, such as patient characteristics, clinical signs and symptoms, and additional examination findings [3]. In addition, parents might have specific expectations about the treatment deemed appropriate for their child, which could influence their acceptance of the physicians decision to (not) prescribe antibiotics [6]. Nowadays, patients’ attitudes towards antibiotic use have become increasingly important since shared decision-making and patient-centered care are seen as key elements for improving quality and safety in healthcare [7]. In order to identify behavioural, cognitive and psychosocial factors underlying parental acceptance of low antibiotic prescription rates, qualitative research methods are needed. Combining qualitative with quantitative research methods potentially results in a more comprehensive understanding of the research problem and associated data, so that the resulting research project will better adress the research questions.

Many countries are now trying to tackle antibiotic resistance by reducing unnecessary or inappropriate prescribing [8]. Strategies to control antibiotic overuse often focus on the initial prescribing decision. Knowledge of the attitude towards antibiotic prescription in a setting where antibiotic overuse is minimal compared with other countries is valuable to guide overuse prevention strategies. Therefore, we performed a mixed methods study assessing the attitude of Dutch parents towards antibiotics for uncomplicated pediatric infectious diseases. Dutch EDs are consulted by 100,000 children every year and over 40% of these consultations are because of an infection [9]. The Netherlands has a universal healthcare system with mandatory private insurance. Most patients with acute illness first consult a general practitioner (GP) before attending the ED. Our primary aim was to explore the opinion of parents on information provided during discharge conversations in the ED, factors that influence their satisfaction with discharge information, and their general opinion on antibiotic use. Secondly, we compared these findings with observed praxis during discharge conversations and we assessed the opinion of pediatricians in the same setting on antibiotic use in children. A specific research question was whether pediatricians adjust the content of their discharge conversation based on the presumed diagnosis, i.e. a viral or bacterial etiology. Because we hypothesized that the provided information could differ between children who did and who did not receive antibiotic treatment, we also aimed to compare the content of discharge conversations between these two patient groups. Together, we report patient-derived and physician-derived factors that contribute to the low prescription rates in our Dutch healthcare setting, in order to extrapolate these to settings where antibiotic overuse is more common.

## Methods

**Fig. 1:**
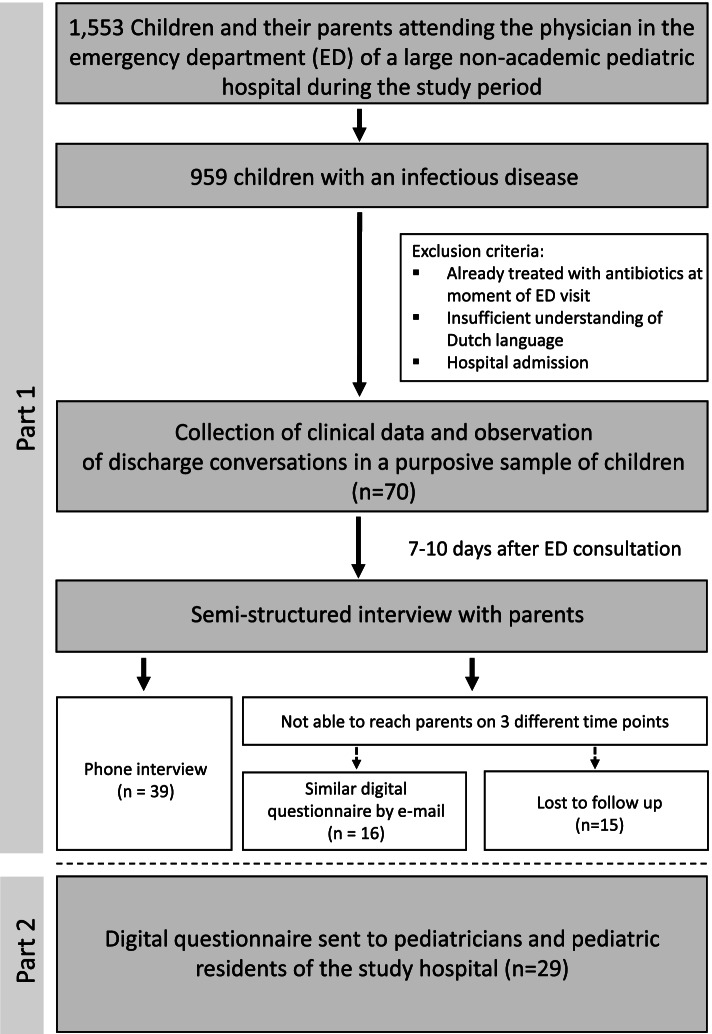
Study design flowchart

Our purpose was to identify patient and physician factors that contribute to the low antibiotic prescription rates in Dutch children and to generate findings that might improve discharge conversations in the ED. We examined discharge conversations in children who consulted the pediatrician at the ED because of a suspected infection. In addition, we explored the attitude of parents and physicians towards antibiotics for uncomplicated pediatric infections in a setting with low antibiotic prescription rates. A convergent mixed method approach with elements of a case study design was chosen. The core principle of mixed methods is that, when used in combination, quantitative and qualitative approaches provide a better understanding of the research question than either approach alone [10]. The quantitative phase included observations of discharge conversations and a survey for physicians, and the qualitative phase included interviews with parents. Figure 1 shows the flowchart of the study.

### Study population and definitions

Eligible subjects were children aged 0–16 years who visited the ED over a period of 2 months (November 26, 2017 – January 26, 2018) because of an infectious disease episode. An infection was defined as a clinical diagnosis made by the physician and was mostly based on the presence of fever or other diagnosis-specific symptoms, such as rhinitis or wheezing. Exclusion criteria were insufficient understanding of the Dutch language, admission to the hospital or initiation of antibiotic treatment before presentation at the ED. The study was conducted in the ED of a large non-academic pediatric hospital in The Netherlands. In 2017, this ED was visited by approximately 13,000 children (0–16 years) of whom 7500 were seen by a pediatrician and of whom 43% was referred by their GP. Referral by the GP is defined as the patient being evaluated and referred by the GP before medical assessment in the ED. In non-academic hospitals in the Netherlands, including our study hospital, pediatricians and pediatric residents do rotating shifts in the ED. The patients were therefore seen by a variety of physicians who all regularly assess children in the ED. Purposeful sampling was used to ensure a representative sample of children with and without antibiotic treatment.

### Data collection

After obtaining informed consent, a member of the study team observed the discharge conversation and filled in a checklist of topics discussed during this conversation. To compose the checklist, we used literature on discharge instructions and the clinical experience of members of our research team [11, 12]. The checklist contained information on the etiology and course of illness, the treatment including antibiotics (explanation of its effect on viral or bacterial infections, indication, duration, dosage, and side effects), and other factors such as follow-up, the opportunity for parents to ask questions, avoidance of medical terminology, and whether the physician checked parental understanding (see Appendix 1 for full checklist details). The definition of ‘safety-netting instructions’ was a doctor-patient conversation about a contingency plan in case specific symptoms (‘red flags’) would occur after discharge. The physician who performed the discharge conversation was informed that the researcher would collect data on the specifics of the infectious disease presentation in order to study the clinical course of the illness. The attending physician was not aware that the researcher was also observing the informative content of the discharge conversation. Seven to 10 days after the ED visit, parents were called for a semi-structured interview. The interview guide was derived from the observation checklist and contained questions regarding the content of and satisfaction with discharge conversations and perceptions about antibiotics in uncomplicated pediatric infections. Most questions were open-ended (Appendix 2). If the study team was unable to contact parents by phone at three different time points, a similar questionnaire was sent by email. Finally, a standardized questionnaire about knowledge, attitude and practices towards antibiotic prescriptions for pediatric infectious diseases, including (uncomplicated) upper respiratory tract infections in particular, was sent to pediatricians and residents working at the study hospital (Appendix 3). This questionnaire was largely based on a questionnaire used for a previous survey in the European Academy of Paediatrics Research in Ambulatory Setting Network [13].

### Data analysis

A specific research question was whether pediatricians adjust their discharge conversation based on the presumed etiology (viral or bacterial), and we hypothesized that the provided information during the discharge conversation would differ between children who received antibiotics and those who did not. Therefore, we tried to achieve a reasonable distribution for this characteristic among our study population. Semi-structured interviews were analysed following the 6-phase thematic analysis as described by Braun and Clarke [14]. We used thematic analysis because this allows the analysis to be driven by the data (by inductive coding) as well as the development of interpretations (themes) that extend understanding beyond just a summary of the data. Interviews were audio recorded and literally transcribed and were coded and themed by using the qualitative data analysis software MaxQDA (v.2018). Two members of the research team performed coding separately. To improve quality of coding and to assure both researchers were in broad agreement, multiple joint meetings to achieve consensus on codes and coding segments were organized during the analytic phase. Following, the two researchers conjointly established key themes. To ensure that all possible themes were identified, two extra researchers were included in the final assignment of themes. The members of the research team included a pediatrician-epidemiologist (MD, PhD, M.H.Sc. Epidemiologist), specifically trained in pediatric infectious diseases and global health with previous experience in qualitative research methodologies, a pediatric infectious diseases subspecialist (MD, PhD), a pediatric resident (MD)/PhD-student with a specific focus on pediatric respiratory tract infections and a medical intern (BSc). There was no inter-observer variability as all observations and interviews were performed by the same researcher. All data was stored in a Castor EDC database. Descriptive analysis of baseline characteristics was performed using IBM SPSS version 24. The results of this mixed methods study were organized by qualitative themes (interviews with parents) and results from the quantitative analyses (observations and questionnaire for physicians) were integrated.

### Ethical considerations

Because this was a non-interventional study, ethical approval was waived. The board committee of the study hospital approved the execution of the study. Parents with legal custody rights provided written informed consent before enrolment. Physicians were asked for their (oral) consent before observing the discharge conversation. Consent for using the survey data was assumed if the physician filled out the questionnaire, which was send with an explanatory text about its purpose.

## Results

### Characteristics of the study population and topics discussed during discharge conversations

We observed a total of 70 discharge conversations by 32 different physicians and 55 parents completed the questionnaire (*n* = 16) or telephone (*n* = 39) follow-up. After including this number of participants no new information arose, therefore reaching theoretical saturation. Children who were lost to follow-up did not differ in age, gender, number of days with fever, presumed etiology of the infection and parents’ educational level from those that were enrolled. Further, 29 physicians (15 pediatricians and 14 pediatric residents) completed a questionnaire including questions around antibiotic prescription choices.

**Fig. 2:**
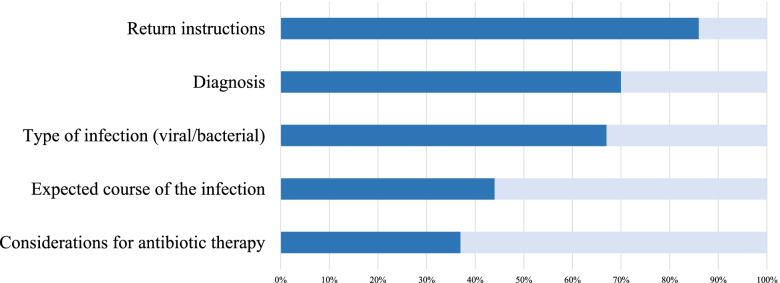
Topics discussed during discharge conversations of 70 children with an infection in the pediatric ED

The mean age of participating children was 2.9 years and the majority suffered from a respiratory or gastro-intestinal infection (Table 1). None of the participants had a serious underlying illness, such as psychomotor retardation or a chronic lung disease. A total of 42 children (60%) received some form of pharmaceutical treatment, and antibiotic treatment was prescribed to 23%. The mean duration of the discharge conversation was 3 min and 44 s (standard deviation ±1 min. 35 s.). Our observations showed that the majority of topics discussed during the discharge conversations were related to the presumed diagnosis, which was discussed in 70% of conversations. During almost all discharge conversations (90%) the physician avoided the use of medical jargon, by using terms such as ‘infection in the lungs’ instead of ‘pneumonia’ or ‘stomach infection’ instead of ‘gastro-enteritis’. Figure 2 provides an overview of topics discussed during discharge conversations.

**Table 1:** Baseline characteristics of 70 children consulting the ED because of a suspected infection

Characteristics of the child and family	
Age of the child; mean (SD), y	2.9 (3.1)
Temperature; n (%)	
< 38.0grC	20 (28.6)
≥ 38.0grC	50 (71.4)
Sex; n (%)	
Boy	40 (57.1)
Girl	30 (42.9)
Siblings; n (%)	
None	51 (72.9)
One or more siblings	19 (27.1)
Ethnicity; n (%)	
Dutch	46 (65.7)
Turkish	12 (17.1)
Moroccan	2 (2.9)
Other	10 (14.3)
Referral to the ED; n (%)	
By general practitioner	62 (88.6)
Self-referral / other	8 (11.4)
Characteristics of the infectious disease	
Suspected pathogen; n (%)	
Viral	49 (70.0)
Bacterial	12 (17.1)
Uncertain	9 (12.9)
Diagnosis; n (%)	
Respiratory tract infection	38 (54.3)
Urinary tract infection	4 (5.7)
Gastro-intestinal infection	15 (21.4)
Skin infection	3 (4.3)
Multiple infections or other	10 (14.3)
Medical treatment; n (%)	
None	28 (40.0)
Antibiotics only	11 (15.7)
Antibiotics and other prescriptions combined	5 (7.1)
Other prescriptions than antibiotics	26 (37.1)

*ED* emergency department; *SD* standard deviation

With respect to the content of the discharge conversations in relation to parental thoughts on information provided during and satisfaction with the conversation, three key themes became evident: 1) parental concerns around (recognizing) disease severity drive their motivation to seek medical care, 2) the importance of safety-netting instructions, and 3) the necessity of feeling reassured for the establishment of trust in the physician’s diagnosis and management plan. With respect to opinions on antibiotic use in uncomplicated infections in children in general, the main theme that became evident was that both parents and physicians experienced a general reluctance to use antibiotics.

### Parental concerns around (recognizing) disease severity drive their motivation to seek medical care

Nearly all patients were referred to the ED by a general practitioner (89%). The most important reason for parents to visit a physician, as mentioned spontaneously by 15 parents, was their anxiousness about the severity of the infection and/or worsening of symptoms. Five parents mentioned that they feared a specific diagnosis, such as pneumonia or a cardiac illness. Other reasons mentioned as considerations in the decision to seek medical care were related to self-empowerment, i.e. the desire to know what parents themselves could do to reduce the child’s symptoms and to be informed of specific symptoms that should trigger them to (re-)visit a health care professional. Parental concerns about the severity of the child’s illness and the capacity to adequately estimate this, were often related to the age of their child (*n* = 12, 22%). Both the anxiety of the presumed increased susceptibility to infections in young children as well as being less anxious about older children were mentioned. Also, two parents spontaneously mentioned that being parents of a firstborn child without siblings caused extra anxiety and was a primary reason to visit a health care professional.

### Parents report the need for specific safety-netting instructions, not for a specific therapy

Overall, follow-up and safety netting instructions were more important for parents than a specific treatment. A total of 31% of parents spontaneously mentioned the importance of the physician telling them they were welcome to come back if the symptoms wouldn’t resolve. An even larger number mentioned they would like to hear specific symptoms and instructions on when and how they should revisit a health care professional (*n* = 44, 80%). This was illustrated by a mother of a 2-year old child with gastro-enteritis: ‘It was nice to receive instructions about when we should be alarmed and to hear that we could always call. This was reassuring and it made me feel at ease that I was allowed to call at any moment’. Similarly, all physicians mentioned that they considered an explanation of alarm symptoms (as part of the safety netting instructions) one of the most important subjects to discuss with parents. In 60/70 (86%) of discharge conversations, safety netting instructions were indeed explicitly discussed. However, safety netting instructions were more often provided when children were not treated with antibiotics (49/54, 91%) than in those cases where an antibiotic was prescribed (11/16, 69%)). Although safety instructions were discussed with the majority of parents, most (*n* = 44) of them expressed a need for (more) details on disease-specific symptoms they should pay attention to. This was illustrated by a mother of a 1-year old child: ‘It is always good to know the alarm symptoms. And the clearer they are, the better we can act on them’.

### Parental trust in the physician’s diagnosis and management is based on a feeling of reassurance and not on the prescribed medical treatment

Reassurance was a key topic mentioned by parents in many (29/55 (53%)) of the interviews. According to parents, a feeling of reassurance can be achieved in several ways. The pediatrician may address this directly, such as by saying that ‘you don’t have to worry’. Feeling reassured and having trust in the pediatricians’ decision also depended on the performed diagnostics. In total, additional laboratory examinations were performed in 26% (18/70) of the included subjects and 39% (7/18) of these parents mentioned spontaneously this made them feel reassured. Trust in the healthcare provider increased if the ED consultation was completely performed by the same physician. Change of staff at any point during the initial assessment, physical exam or discharge conversation decreased parents’ feeling of trust and reassurance or caused confusion, as mentioned by five parents. In addition, previous experiences with the physician or the hospital determined the extent to which parents felt trust. Four parents who spontaneously mentioned they had negative experiences with the GP, also mentioned that they trusted their attending ED physician more than the GP. None of the parents mentioned specific outcomes, such as whether or not antibiotics were prescribed, as a factor influencing their trust in the physician's treatment plan. This is further emphasized by the observation that 35% of parents spontaneously mentioned they would rather rely on the physician’s assessment than unnecessarily give an antibiotic to their child.

### General reluctance to use antibiotics is felt by both parents and physicians, but the basis for this reluctance differs

Although most parents consulted a pediatrician because they were anxious about the severity of the infection, 65% spontaneously mentioned they wanted their child to fight off the infection ‘naturally’ and didn’t necessarily prefer drugs over a ‘wait and see’ approach. This was illustrated by a parent of a 16-month-old child with an upper respiratory tract infection: ‘The body should be able to heal all sorts of things by itself. So if it’s possible without antibiotics, then we’d rather go without’. In case the physician did decide to prescribe medication, 86% of parents mentioned that they did not necessarily expect to be prescribed antibiotics. Other drugs to relieve their child’s complaints, such as antipyretics, were also sufficient to satisfy parents. One of parents’ main drivers for not wanting antibiotics, was the fear for negative side effects.

**Fig. 3:**
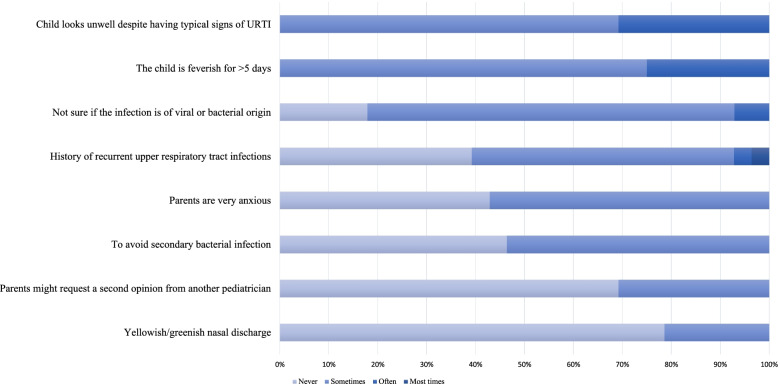
Reasons of physicians to prescribe antibiotic treatment in children with an upper respiratory tract infection (URTI)

Physicians also reported a general reluctance to prescribe an antibiotic agent. Both pediatricians and residents mentioned they prescribe antibiotics in a minority of the respiratory infectious disease cases they assess (median 14%). In general, physicians were reluctant to prescribe antibiotic agents, which was also experienced by parents. An example was a mother of a 3 week old boy with an upper respiratory tract infection who told us about her experience with her other children: ‘I’ve never experienced that a physician directly prescribes antibiotics. They have to be really certain that it’s caused by a bacterium’. Most physicians consider antibiotic treatment for children with respiratory tract infections based on clinical characteristics, as illustrated in Fig. 3. Interestingly, parental anxiety (57%) or the thought that parents might otherwise go elsewhere to seek a second opinion (31%) were also mentioned as reasons to sometimes prescribe antibiotics. This indicates that parental demands and perceptions are of importance in physician’s treatment considerations.

Information about adequate indications for antibiotic treatment can be discussed during the discharge conversation. In our study, the indication for antibiotic treatment was mostly discussed when antibiotics were prescribed: 13/16 parents (81%) who were given a prescription for antibiotics, were provided with explanation on the choice for antibiotic treatment, while only 8/54 (15%) of parents of children that were not treated with antibiotics, received any form of explanation about the (absent) indication for antibiotics. In 47/70 (67%) of all cases, the physician explained whether he or she suspected a viral or bacterial infection. However, parents don’t seem to use this information to form their opinion on whether they prefer antibiotic therapy or not. The most important reason for parents to potentially prefer antibiotics, as mentioned by 23 of them (42%), was the idea that this treatment could reduce or shorten their child’s symptoms, especially if the prolonged duration of symptoms was one of the main reasons to seek medical care. Fear of AMR did not seem to motivate a majority of parents to oppose antibiotic treatment, since this was only spontaneously mentioned by three parents (5%). In contrast, almost all physicians (97%) were concerned that misuse of antibiotics would lead to increased AMR.

## Discussion

In this mixed methods study we explored parents’ reasons to visit the ED, factors that influenced their satisfaction with discharge information and their attitude towards antibiotic treatment for pediatric infections in general. We compared the views of parents with observations of discharge conversations during ED visits and opinions of pediatric residents and pediatricians.

Children with febrile illnesses are among the biggest patient groups receiving unnecessary antibiotics. The prescription rate for children without comorbidity who suffer from upper respiratory tract infections in hospitals in Europe varies from 15 to 67%, the Netherlands being among the countries with the lowest prescription rates [3]. In our study population of Dutch parents visiting the ED with a child with infectious disease symptoms, over 80% explicitly mentioned that even if pharmaceutical treatment is indicated to cure their child, they do not prefer antibiotics over other types of medication. While previous literature shows that in other European countries and the USA most parents (50–74%) expect or want antibiotics in case of a cold and fever until roughly 15 years ago [15–17], this practice has been changing with increasing awareness of AMR [18]. We show that a general reluctance towards antibiotic use among both parents and physicians contributes to the low antibiotic prescription rates in our Dutch setting. The hesitance of parents to use antibiotics is fuelled by the idea that children should be able to fight off infections ‘naturally’. Instead of therapeutic treatment, a ‘wait-and-see’ strategy and awareness of alarm symptoms should be sufficient for the majority of pediatric febrile illnesses [19]. Nevertheless, fever phobia is a common phenomenon among parents worldwide: 53–99% of parents worry about harmful effects of fever [20–24]. However, Dutch parents seem to be less distressed if their child has a fever. A questionnaire among Dutch parents showed that only one in five Dutch parents worry about harmful effects of fever in general, and 13–37% worried about specific consequences such as brain damage, dehydration or convulsions [25]. These differences, in combination with our findings, suggest a different mind-set in Dutch parents. Several factors could underlie this difference in parental attitude. In the Netherlands, and most other high income countries, child mortality is low and only a small fraction of pediatric febrile illnesses is caused by serious bacterial infections. This is in sharp contrast with low- or middle-income countries, where children are at greater risk to die from infectious diseases [26]. Also, the Dutch National Immunisation Programme is extensive, healthcare facilities are easily accessible and the government provides information on viral infections and on proper indications for antibiotics via commercials on national television and radio [27]. In some high-income countries, other factors might explain the differences in attitude towards antibiotics. For example, a study in the USA showed that physicians and pediatricians frequently believe that parents expect antibiotics and that it’s too late to change this expectation once the patient visits the doctor’s office. Also, physicians fear a negative review or believe parents will seek medical care and an antibiotic prescription elsewhere [28]. These expectations seem to be different from those of Dutch physicians, the majority of whom did not report such expectations or concerns.

The factors underlying a reluctance to antibiotic use differed between parents and physicians. Physicians were mostly concerned that the misuse of antibiotics could lead to increased AMR. Previous studies in other settings show that most parents know or worry about the risks of AMR development [16, 29–31]. However, Dutch parents didn’t seem to be motivated by the fear of AMR. Parents were mostly afraid of direct negative side effects of antibiotics and wanted to minimize the number of prescriptions for their children in general. A medical assessment and reassurance about the health status of their child was more important than receiving any form of treatment, including antibiotics. This is in line with a previous Dutch study [25] and further emphasized by our finding that the possibility of antibiotic treatment wasn’t even mentioned in the majority of discharge conversations with parents of children that did not receive antibiotics.

Previous studies show that structured and complete discharge instructions are associated with increased parental satisfaction [12, 32]. In our study, parents also stated to feel more satisfied if they received complete information, including a diagnosis, expected course of disease, safety netting advice and an explanation of the treatment plan. The discharge conversations could be improved, since only a small number of parents received information on all these topics. Specifically, the expected course of the infection and the consideration for antibiotics were addressed less often. According to both parents and physicians, information on return instructions was the most important topic during the discharge conversation. Although this topic was discussed most frequently during our observations, parents reported a need for more details. Since parents’ motivation to seek help was related to the concern about their inability to assess their child’s health status, paying attention to alarm symptoms could help them recognize health changes. Other studies show that parents don’t recall (57–78%) or don’t recognize (70%) previously mentioned alarm symptoms after being admitted to the ED [12, 33, 34]. While these studies emphasize the need for detailed and structured information on how to recognize alarm symptoms, this also suggests that part of the information given during a discharge conversation will be lost. Interestingly, we observed that return instructions were discussed more frequently if a child wasn’t prescribed antibiotics. An explanation for this observation could be that physicians consider antibiotic treatment to be the ‘safe choice’. Physicians may be more cautious when refraining from antibiotic treatment and therefore pay more attention to providing these instructions to parents. Extensive safety netting may be an alternative for (unnecessary) use of antibiotics in our setting and elsewhere. In order to improve the overall quality of the provided information during the discharge conversations, physicians might benefit from a checklist of the essential topics. Also, parents may feel reinforced by a similar checklist, because it could help them to ask for (extra) information if not provided directly.

To our knowledge this is the first study that combines parental views on antibiotics and discharge information given during ED visits with the opinion of physicians on these topics and with observations of discharge conversations. By doing so, we were able to examine the factors underlying the reluctance towards antibiotic use in the unique Dutch setting. Because this was a single centre study, its main limitation is the generalizability to other hospitals or settings, such as middle- or low-income countries in which healthcare facilities are less accessible, immunization coverage is lower and infectious diseases prevalence rates are different [35]. Also, our observations of the discharge conversations may be an underestimation of what was actually discussed with parents, because we did not observe the complete medical consultation and therefore we could’ve missed information that was discussed before the discharge conversation. Further, physicians might have adjusted their discharge instructions because a researcher was present. We tried to minimize this effect by not informing them about the objectives of the observations. Finally, because a subgroup of parents was followed up with a digital questionnaire instead of an interview, the quality of data collection in this group could have been less compared with parents that underwent an interview as part of the follow up.

## Conclusion

The Netherlands has one of the lowest antibiotic prescription rates for pediatric febrile illnesses. With our mixed methods approach, we showed that a reluctance in both pediatricians and parents towards antibiotic use exists. While the physician’s focus is on the correctness of the indication of the antibiotics and on the prevention of AMR, parents prefer a thorough medical assessment over any type of medical treatment, including antibiotics. Safety-netting advice is the most important topic to discuss during the discharge conversation, according to both parents and pediatricians. Detailed return instructions might be an alternative for antibiotic overuse in the Dutch ED setting. Since this is an easy to implement strategy, putting emphasis on safety netting in conversations with parents of children with infectious diseases might also be of benefit in other settings. This requires the education of physicians on the importance of and the need for detailed instructions on parental recognition of alarm symptoms.

## Supplementary Information

**Additional file 1.**

## Data Availability

The datasets generated and/or analysed during the current study are not publicly available but are available from the corresponding author upon reasonable request.

## Abbreviations

AMR: Antimicrobial resistance
ED: Emergency department
GP: General practitioner
