# Supplementary material for: Parental and physician’s point-of-view towards antibiotic prescriptions and discharge conversations in the pediatric emergency department
Source: BMC Pediatr. 2022 Mar 10;22:121. doi: 10.1186/s12887-022-03173-w (PMC8908586; doi:10.1186/s12887-022-03173-w)
Supplement: Supplementary file 1 — Additional file 1. [file 12887_2022_3173_MOESM1_ESM.docx]

**Appendix 1 Checklist observations**

| Topic | Discussed? | Specification |
| --- | --- | --- |
| **Disease-specific information** | | |
| Diagnosis | □ Yes □ No |  |
| Expected course of infections | □ Yes □ No |  |
| Type of infection (viral or bacterial) | □ Yes □ No |  |
| Instructions on when to re-contact a physician | □ Yes □ No |  |
| Follow-up | □ Yes □ No |  |
| Treatment | □ Yes □ No |  |
| **Treatment with antibiotics** | | |
| Indication for antibiotics for this consultation | □ Yes □ No |  |
| How antibiotics work | □ Yes □ No |  |
| Difference of effect of antibiotics on viral and bacterial infections | □ Yes □ No |  |
| Importance of finishing the antibiotic regimen | □ Yes □ No |  |
| Specifics of the antibiotic treatment | How many times a day: □ Yes □ No  Doses in mg: □ Yes □ No  Dosage in ml: □ Yes □ No  Duration: □ Yes □ No Specification:  Side effects: □ Yes □ No Specification: | |
| **General** | | |
| Physician checks parental understanding | □ Yes □ No |  |
| Physician provides the possibility for parents to ask questions | □ Yes □ No |  |
| Avoidance of medical terminology | □ Yes □ No |  |
| Duration of discharge conversation | …… min and ….. seconds | |

**Appendix 2 Interview guide**

**Question 1:** What has been the course of the infection since the discharge conversation on the Emergency Department (ED)?

**Question 2:** What are your thoughts about antibiotics as treatment for uncomplicated infections in children?

**Question 3a:** If an antibiotic treatment was prescribed:

- Can you explain why the doctor prescribed an antibiotic treatment for your child?
- Can you explain how you should administer the antibiotic treatment (frequency/dosage/amount, etc.)?
- Were there any difficulties in administering the antibiotic treatment?
  - If yes: What were difficulties in the administration of the antibiotic treatment?
- Did you complete the antibiotic treatment?
  - If not: Why did you not finish the treatment?
- Did your child experience any side effects?
  - If yes: Which side effects did your child experience?

**Question 3b:** If there was no antibiotic prescribed:

- Why didn’t the doctor prescribe antibiotic treatment for your child?
- What is your opinion on the fact that the doctor did not prescribe antibiotic treatment for your child?
- Did you visit another health care professional after the visit to the ED?
  - If yes: Why? What was the result?
  - If no: Why not? In retrospect, would you have rather done that?
- After the visit to the Emergency Department, did your child receive antibiotic treatment?
  - If yes: What was the indication for the antibiotic treatment?
  - If yes: Who prescribed the antibiotic treatment?
  - If no: In how many days was your child recovered?

**Question 3c:** If there was delayed antibiotic prescribing:

- Can you explain why the doctor prescribed an antibiotic treatment for your child?
- Did you give the antibiotic treatment?
  - If yes:
    - After how many days after discharge from the ED did you give the antibiotic treatment?
    - Can you explain how you should administer the antibiotic treatment (frequency/dosage/amount, etc.)?
    - Were there any difficulties in administering the antibiotic treatment?
    - Did you complete the antibiotic treatment?
      - If not: why did you not finish the treatment?
    - Did your child experience any side effects?
      - If yes: which side effects did your child experience?
  - If no: Why did you not give the antibiotic treatment?

**Question 4a:** What was your opinion on the discharge conversation on the ED?

**Question 4b:** What was your opinion on the quality of discharge conversation on the ED? (Very good, good, not good/not bad, bad, very bad)

**Question 4c:** What are important topics that should be discussed during discharge conversations on the ED when a child is suspected of an infection?

**Question 4d:** How can we improve the quality of the discharge conversations on the ED?

**Appendix 3 Questionnaire for physicians**

| **1. What is your age (in years)?** | | | | | | | | | |
| --- | --- | --- | --- | --- | --- | --- | --- | --- | --- |
|  | | | | | | | | | |
| **2. Tell us your specialty, please.** | | | | | | | | | |
|  | ⬜ | Pediatrician |  |  | | | | | |
|  | ⬜ | Pediatric resident |  |  | | | | | |
|  | ⬜ | Medical student |  |  | | | | | |
|  | | | | | | | | | |
| **3. What is your gender?** | | | | | | | | | |
|  | ⬜ | Female | ⬜ | Male | | | | | |
|  | | | | | | | | | |
| **SECTION A – We present you three statements. Please tell us your opinion** | | | | | | | | | |
|  | | | | | Strongly Agree | Agree | Uncertain | Disagree | Strongly disagree |
| **4. The vast majority of URTIs resolve without antibiotic use** | | | | | ⬜ | ⬜ | ⬜ | ⬜ | ⬜ |
| **5. Unnecessary antibiotic use drive antimicrobial resistance** | | | | | ⬜ | ⬜ | ⬜ | ⬜ | ⬜ |
| **6. Acute Otitis Media is best treated with first line/narrow spectrum antibiotics when necessary.** | | | | | ⬜ | ⬜ | ⬜ | ⬜ | ⬜ |

| **SECTION B – Would you consider prescribing antibiotics to a child with symptoms of upper respiratory tract, in the following cases?** | | | | | | | | | |
| --- | --- | --- | --- | --- | --- | --- | --- | --- | --- |
|  | | | | | Always | Most times | Often | Some-times | Never |
| **7. To avoid secondary bacterial infection** | | | | | ⬜ | ⬜ | ⬜ | ⬜ | ⬜ |
| **8. Because you are not certain if the infection is of viral or bacterial origin** | | | | | ⬜ | ⬜ | ⬜ | ⬜ | ⬜ |
| **9. Because the child is feverish for >5 days** | | | | | ⬜ | ⬜ | ⬜ | ⬜ | ⬜ |
| **10. Because there is a past history of recurrent upper respiratory tract infections** | | | | | ⬜ | ⬜ | ⬜ | ⬜ | ⬜ |
| **11. Because child has yellowish/greenish nasal discharge** | | | | | ⬜ | ⬜ | ⬜ | ⬜ | ⬜ |
| **12. Because parents are very anxious** | | | | | ⬜ | ⬜ | ⬜ | ⬜ | ⬜ |
| **13. Because parents might request a second opinion from another paediatrician** | | | | | ⬜ | ⬜ | ⬜ | ⬜ | ⬜ |
| **14. Because the child looks unwell despite having typical signs of upper respiratory tract infection** | | | | | ⬜ | ⬜ | ⬜ | ⬜ | ⬜ |
| **15. What do you think the benefit will be of prescribing antibiotics to a child with acute otitis media?** | | | | | | | | | |
| **There is a benefit** | | ⬜ | Yes | ⬜ | No | | | |  |
| **If yes:** | | **Reduction in days of fever (for how many days)?** | | | | | | | |
|  | | ⬜ | No reduction | | | | | | |
|  | | ⬜ | 1 day | | | | | | |
|  | | ⬜ | 2 days | | | | | | |
|  | | ⬜ | 3 days | | | | | | |
|  | | ⬜ | 4 days | | | | | | |
|  | | **Reduction in days of pain (for how many days)?** | | | | | | | |
|  | | ⬜ | No reduction | | | | | | |
|  | | ⬜ | 1 day | | | | | | |
|  | | ⬜ | 2 days | | | | | | |
|  | | ⬜ | 3 days | | | | | | |
|  | | ⬜ | 4 days | | | | | | |
|  | | **Less chance of developing mastoiditis.** | | | | | | | |
|  | | ⬜ | Yes | | | | | | |
|  | | ⬜ | No | | | | | | |
| **16. The number of children with acute otitis media I need to give antibiotics to prevent one case of mastoiditis is:** | | | | | | | | | |
| ⬜ | 5 | ⬜ | 5,000 | | | | | | |
| ⬜ | 50 | ⬜ | 50,000 | | | | | | |
| ⬜ | 500 | ⬜ | 500,000 | | | | | | |

| **17. What do you think the benefit will be of prescribing antibiotics to a 5 years old child with pharyngitis?** | | | | | | | | | | |
| --- | --- | --- | --- | --- | --- | --- | --- | --- | --- | --- |
| **There is a benefit** | | | ⬜ | | Yes | | | ⬜ | No |  |
| **If yes:** | | | **Reduction in days of fever (for how many days)?** | | | | | | | |
|  | | | ⬜ | | No reduction | | | | | |
|  | | | ⬜ | | 1 day | | | | | |
|  | | | ⬜ | | 2 days | | | | | |
|  | | | ⬜ | | 3 days | | | | | |
|  | | | ⬜ | | 4 days | | | | | |
|  | | | **Reduction in days of pain (for how many days)?** | | | | | | | |
|  | | | ⬜ | | No reduction | | | | | |
|  | | | ⬜ | | 1 day | | | | | |
|  | | | ⬜ | | 2 days | | | | | |
|  | | | ⬜ | | 3 days | | | | | |
|  | | | ⬜ | | 4 days | | | | | |
|  | | | **Less chance of developing rheumatic fever.** | | | | | | | |
|  | | | ⬜ | | Yes | | | | | |
|  | | | ⬜ | | No | | | | | |
|  | | | **Less chance of developing peritonsilar abscess.** | | | | | | | |
|  | | | ⬜ | | Yes | | | | | |
|  | | | ⬜ | | No | | | | | |
| **18. The number of children I need to give antibiotics to prevent one case of peritonsillar abscess is:** | | | | | | | | | | |
| ⬜ | 5 | | ⬜ | | 5,000 | | | | | |
| ⬜ | 50 | | ⬜ | | 50,000 | | | | | |
| ⬜ | 500 | | ⬜ | | 500,000 | | | | | |
| **The right answers in questions are highlighted in yellow.* | | | | | | | | | | |
| **Section C** | | | | | | | | | | |
| **19.1 How many children do you usually examine during the winter months (December-February)?** | | | | | | | | | | |
|  | | | | | | | | | | |
| **19.2 How many antibiotic prescriptions do you usually give every day for children during the winter months (December‐February)?** | | | | | | | | | | |
|  | | | | | | | | | | |
| **20. What percentage of these daily antibiotic prescriptions are for upper respiratory tract infection?** | | | | | | | | | | |
|  | | | | | | | | | | |
| **21. Do you perform rapid antigen testing (strep test) before prescribing antibiotics on a child with tonsillitis?** | | | | | | | | | | |
| ⬜ | | Yes | | ⬜ | | No | | | | |
|  | | | | | | | | | | |
| **22. What is the age group that receives the most antibiotics at your practice?** | | | | | | | | | | |
| ⬜ | 0‐3 months | | | ⬜ | | | 6‐8 years | | | |
| ⬜ | 4‐11 months | | | ⬜ | | | 9‐15 years | | | |
| ⬜ | 1‐5 years | | |  | | |  | | | |

| **23. Please rank the following six indications: Prescribing antibiotics most commonly (rank 6) and least**  **commonly (rank 1). Please assign ranks 2 to 5 to the other four indications.** | | | | | | | | |  |
| --- | --- | --- | --- | --- | --- | --- | --- | --- | --- |
| Common Cold |  |  | | | | | | |  |
| Pharyngitis |  |  | | | | | | |  |
| Tonsilitis |  |  | | | | | | |  |
| Acute Otitis Media |  |  | | | | | | |  |
| Sinusitis |  |  | | | | | | |  |
| Bronchitis |  |  | | | | | | |  |
| **24. How often would you prescribe antibiotics for the following indications:** | | | | | | | | |  |
|  | | | Always | Most times | Often | Some-times | Never |  | |
| Common Cold | | | ⬜ | ⬜ | ⬜ | ⬜ | ⬜ |  | |
| Pharyngitis | | | ⬜ | ⬜ | ⬜ | ⬜ | ⬜ |  | |
| Tonsilitis | | | ⬜ | ⬜ | ⬜ | ⬜ | ⬜ |  | |
| Otitis media | | | ⬜ | ⬜ | ⬜ | ⬜ | ⬜ |  | |
| Purulent rhinitis | | | ⬜ | ⬜ | ⬜ | ⬜ | ⬜ |  | |
| Sinusitis | | | ⬜ | ⬜ | ⬜ | ⬜ | ⬜ |  | |
| Bronchitis | | | ⬜ | ⬜ | ⬜ | ⬜ | ⬜ |  | |

|  | | | | | Always | Most times | Often | Some-times |  | Never |
| --- | --- | --- | --- | --- | --- | --- | --- | --- | --- | --- |
| **25. How often do you explain to the parents the reasons for which you prescribe antibiotics?** | | | | | ⬜ | ⬜ | ⬜ | ⬜ |  | ⬜ |
| **26. How often do you talk to the parents about the risks and problems of taking antibiotics?** | | | | | ⬜ | ⬜ | ⬜ | ⬜ |  | ⬜ |
| **27. Do you give the parents any leaflets about the risks and benefits of antibiotics?** | | | | | ⬜ | ⬜ | ⬜ | ⬜ |  | ⬜ |
| **28. How often do parents request on antibiotic prescription?** | | | | | ⬜ | ⬜ | ⬜ | ⬜ |  | ⬜ |
| **29. How often would you consider prescribing an antibiotic for an infection or presumed viral etiology?** | | | | | ⬜ | ⬜ | ⬜ | ⬜ |  | ⬜ |
| **30. How often do parents disagree with your decision to prescribe antibiotics?** | | | | | ⬜ | ⬜ | ⬜ | ⬜ |  | ⬜ |
| **31. How often do parents disagree with your decision NOT to prescribe antibiotics?** | | | | | ⬜ | ⬜ | ⬜ | ⬜ |  | ⬜ |
| **32. How often do you think parents wish to have antibiotics without saying so?** | | | | | ⬜ | ⬜ | ⬜ | ⬜ |  | ⬜ |
|  | | | | |  |  |  |  |  |  |
| **33. Have you ever heard of delayed antibiotic prescribing?** | | | | | | | | | | |
| ⬜ | | Yes | ⬜ | No —> if no, end of questionnaire | | | | | | |
| **34. If yes, do you ever practice delayed antibiotic prescribing?** | | | | | | | | | | |
| ⬜ | Yes | | ⬜ | No |  |  |  |  |  |  |

| **35 What are important points that you discuss with parents when their child, suspected of an infection, is going to be discharged from the emergency department?** |
| --- |
|  |
| **36 What are important points that you discuss with parents when an antibiotic is prescribed in order to treat an infection when their child is discharged from the emergency department?** |
|  |
| **37 What do you often forget to explain when discharging a child from the emergency department when the child is suspected of an infection?** |
|  |

This questionnaire was largely based on a questionnaire designed by members of the European Academy of Paediatrics Research in Ambulatory Setting Network. Reference: Grossman Z, del Torso S, Hadjipanayis A, van Esso D, Drabik A, Sharland M. Antibiotic prescribing for upper respiratory infections: European primary paediatricians' knowledge, attitudes and practice. Acta Paediatr. 2012;101(9):935-40.
